# High-Fish Oil and High-Lard Diets Differently Affect Testicular Antioxidant Defense and Mitochondrial Fusion/Fission Balance in Male Wistar Rats: Potential Protective Effect of ω3 Polyunsaturated Fatty Acids Targeting Mitochondria Dynamics

**DOI:** 10.3390/ijms20123110

**Published:** 2019-06-25

**Authors:** Vincenzo Migliaccio, Raffaella Sica, Ilaria Di Gregorio, Rosalba Putti, Lillà Lionetti

**Affiliations:** 1 Department of Chemistry and Biology “Adolfo Zambelli”, University of Salerno, 84084 Fisciano, Italy; vincenzo.migliaccio@unina.it (V.M.); idigregorio@unisa.it (I.D.G.); 2 Department of Biology, University of Naples, Federico II, 80126 Naples, Italy; raffaella.sica@tiscali.it (R.S.); rosalba.putti@unina.it (R.P.)

**Keywords:** ω3 polyunsaturated fatty acids, testis, oxidative stress, apoptosis, MFN2, DRP1

## Abstract

High-fat diets rich in fish oil (HFO diet, mainly ω3-PUFAs), in contrast to high-fat diets rich in lard (HL diet, mainly saturated fatty acids) have been shown to induce improvement in mitochondrial function and fusion processes associated with a reduction in reactive oxygen species production in both liver and skeletal muscle. High-fat diets may also impair testicular function, and mitochondria represent important cellular organelles with a pivotal role in reproductive function. Mitochondria are dynamic organelles that frequently undergo fission/fusion processes. A shift toward mitochondrial fusion process has been associated with improvement of mitochondrial function, as well as with ω3-PUFAs protective effects. The present study aimed to analyze the effect of chronic overfeeding (six weeks) with HFO or HL diet on testicular tissue histology, oxidative stress, antioxidant defenses, and mitochondrial fusion (mitofusin 2) and fission (dynamic related protein 1) protein. Our results showed that HFO diet induced less testicular histology impairment, oxidative stress, and apoptosis compared to a HL diet. This finding was associated with an increase in antioxidant activities and a shift toward mitochondrial fusion processes induced by HFO diet compared to HL diet, suggesting that ω3-PUFAs may act as bioactive compound targeting mitochondria dynamics to prevent testicular impairment.

## 1. Introduction

Dietary fats represent the main source of excess energy intake associated with both obesity and metabolic disorders in modern society [1]. Food lipid composition, in terms of fatty acids, is characterized by two big families: Saturated fatty acids (SFA) and unsaturated fatty acids (UFA), which include monounsaturated and polyunsaturated fatty acids. Diverse dietary fat sources differently affect cellular metabolism, with different effects on metabolic disease risks. For example, it is well-known that omega 3 polyunsaturated fatty acids (ω3-PUFAs), mainly by fish consumption, play a key role in cardiovascular diseases prevention [2,3,4,5,6], whereas SFAs, mainly consumed by food animal sources such as meat and lard, have been associated with increased risks for cardiovascular health [7,8,9]. It has been suggested that ω3-PUFAs are the most important bioactive lipids, providing health benefits either through their effects on cell membrane structure and/or through modulating the expression of gene by regulating transcription factors related to energy supply and cell cycle [6,10,11,12,13,14]. Indeed, ω3-PUFAs, due to the long tail of double bonds, help to keep membrane flexibility and fluidity, maintaining normal cell homeostasis and cell-to-cell communication. ω3-PUFAs have also been suggested to act as bioactive compounds, targeting mitochondria, improving mitochondrial function and dynamic behavior, and therefore, counteracting cellular damage, tissue dysfunction, and metabolic diseases. Studies in rodents and humans showed that ω3-PUFAs can reduce obesity and related diseases by reducing fat deposition and stimulating mitochondria fat oxidation and fusion processes [13,15,16,17,18]. Mitochondria are dynamic organelles that frequently undergo fission/fusion processes to remodel the mitochondrial network in accordance with cellular energy requirements: Mitochondrial fusion of individual mitochondria into dynamic network can induce improvement in mitochondrial function, whereas mitochondrial fission is useful to remove damaged mitochondria through mitophagy [19,20,21]. A shift toward fission process has been associated with mitochondrial dysfunction and metabolic disease induced by SFAs. On the other hand, fusion process, associated with ameliorated mitochondrial function, could be a key mechanism for several positive effects induced by ω3-PUFAs [12,21,22,23]. Indeed, ω3-PUFAs have been shown to improve skeletal muscle insulin sensitivity and inflammation [12,13], in association with a shift toward mitochondrial fusion phenotype in rats fed a high-fish oil (HFO) diet compared to rats fed a high-lard (HL) diet [24]. On the other hand, reduced skeletal muscle insulin sensitivity has been found to be associated with a shift toward mitochondrial fission with reduction of fusion protein, mainly mitofusin 2, in obesity and insulin resistance experimental model [25,26,27]. Moreover, several in vitro and in vivo experiments also showed that ω3-PUFAs induced improvement in mitochondrial function and fusion processes, as well as reduction in reactive oxygen species (ROS) production in liver. Mitochondrial ROS have been known to cause apoptosis, oxidative stress, and aging [28]. EPA and DHA increased Mfn2 expression and ATP levels, and decreased oxidative stress in an in vitro steatotic hepatocyte model [29]. Moreover, we previously showed that HFO, in contrast to HL diet, elicited improvement in mitochondrial function and fusion processes associated with reduction in ROS in liver from male Wistar rats [30]. In the same experimental model, the replacement of lard with fish oil attenuated the development of obesity, as well as systemic and tissue inflammation, ameliorating histological features in different tissues, such as white adipose tissue, skeletal muscle, and liver, suggesting that the protective effect of ω3-PUFAs against metabolic disease relies on overlapping mechanisms in diverse peripheral tissues [31]. Altogether, these results indicated that HFO diets were less dangerous in targeting mitochondria than HL diets during chronic overfeeding.

The aim of the present study was to extend our previous work to another tissue, namely the testicular tissue, that can be damaged by high-fat diet impairing reproductive function [32]. Several studies have been carried out to shed light on the effects of fat overnutrition on male fertility. Low ω3-PUFAs and high SFA levels have been shown to be negatively correlated with altered sperm fatty acid profiles in human spermatozoa [33], as well as with reduction in fertilization capacity [34,35]. The positive effects of ω3-PUFAs in dietary patterns on male fertility parameters and fecundability were recently reviewed [36]. Castillo and coworkers showed that ω3-PUFAs supplementation played a protective role in rat testis by reducing oxidative damage caused by intermittent hypobaric hypoxia through the reinforcement of antioxidant defense system [37]. Noteworthy, infertility may be included among the physiological dysfunction induced by impairment of cellular antioxidant system, including both mitochondrial and cytosolic antioxidant enzymes. Regarding this aspect, mitochondria represent important cellular organelles with a pivotal role in reproductive function, as they represent the principal site of ROS generation and provide energy to support cellular maturation and differentiation during spermatogenesis, as well as sperm motility, capacitation, and fertilizing ability [38]. However, the exact role of sperm mitochondria is especially controversial, with a key role of mitochondria-generated ROS function in signaling and apoptotic pathway [39]. Castillo et al. showed that ω3-PUFAs induced increases in both superoxide dismutase (SODs) and glutathione peroxidase (GPx) activities, suggesting that these enzymatic activities mainly acted as homeostatic mechanisms to counteract the increased oxidative stress [37]. In accordance, other researchers demonstrated that a pre-treatment with ω3-PUFAs reduced doxorubicin-induced oxidative damages and apoptosis in testis by increasing antioxidant system activity and improving tissular tissue morphology [40]. Most recently, in vivo analyses showed that a high SFA diet (34.9% fat) induced testicle stress and Sertoli cells apoptosis in association with increase in ROS levels. Moreover, the same authors demonstrated that ω3-PUFAs supplementation in vitro protected Sertoli cells from the harmful effect of palmitic acid, preventing cellular oxidative stress and apoptosis [41]. In our recent work, we found that antioxidant activity plays a key role in the control of cellular stress, apoptosis and tissular damage in rat testis [42]. In fact, we showed that high SFA diet (45% fat) negatively affected antioxidant system in rat testis by inducing malondialdehyde (MDA) accumulation as a product of lipid peroxidation and apoptosis. In the same experimental work, simultaneous exposure to high fat diet and environmental pollutants elicited a further rise in oxidative stress and apoptosis, associated with a worsened antioxidant system with a strong reduction in both SODs and GPx activities [42]. Therefore, spermatogenesis was compromised.

Considering the contribution of mitochondria to ROS production as well as the emerging role of mitochondrial dynamics in cell homeostasis, in the present work, we focused on the effect of different dietary fat source, namely lard (HL diet) or fish oil (HFO diet), during chronic high-fat feeding (six-week period) on testis histological features and mitochondrial performance by assessing oxidative stress defense markers and mitochondrial dynamic protein contents in male Wistar rats. Testis morphology was analyzed to evaluate possible presence of tissue alterations. Oxidative stress and antioxidant defenses were assessed by measuring the level of MDA and the activity of the main antioxidant enzymes activities, namely SODs and GPx activities. The content of the pro-apoptotic protein Bcl-2-associated X protein (BAX) was determined by western blotting together with caspase 3 activity to evaluate the trigger of apoptosis. Finally, the effect of HFO vs. HL diet on mitochondrial dynamics was assessed by analyzing the amount of dynamin-related protein 1 (DRP1), the main protein involved in fission process, and mitofusin 2 (MFN2), the main protein involved in fusion process. The present result showed a positive effect of HFO diet on mitochondrial fusion/fission balance with a shift toward mitochondrial fusion in association with reduction in oxidative stress in testis. Therefore, the present work confirmed that ω3-PUFAs may act as bioactive compound targeting mitochondria and that mitochondrial fusion/fission balance may be involved in the mechanisms by which ω3-PUFAs exert their benefit in different tissues, contributing to the maintenance of cellular homeostasis and overall health.

## 2. Results

### 2.1. Different Impact of HL and HFO Diet on Body Weight Gain and Testis Weight and Morphology

**Figure 1:**
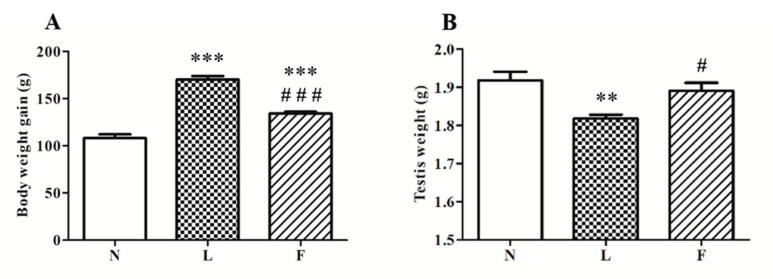
High lard (HL) and high fish oil (HFO) diets differently affect body weight gain (**A**) and testicular weight (**B**). Values are represented graphically as means ± standard error of the mean (SEM) of 8 different animals for each group. Significant differences are shown: ** *p* < 0.01 vs. N; *** *p* < 0.001 vs. N; # *p* < 0.05 vs. L; ### *p* < 0.001 vs. L.

At the end of the treatment period, body weight gain was calculated as difference between final body weight and initial body weight for each animal. In accordance with literature data, high-fat feeding induced an increase in body weight gain compared to standard diet. In particular, HL diet fed rats (L rats) showed the highest body weight gain compared to control (N rats, ~57%) and HFO fed rats (F rats, ~20%). On the other hand, F rats showed increased body weight gain vs. N (~25%), whereas they showed significantly lower body weight gain increases compared to L rats (Figure 1, A). Moreover, we showed a slight reduction in testis weight in L rats (~6%) vs. N, whereas F rats exhibited testis weight similar to control rats (Figure 1B).

**Figure 2:**
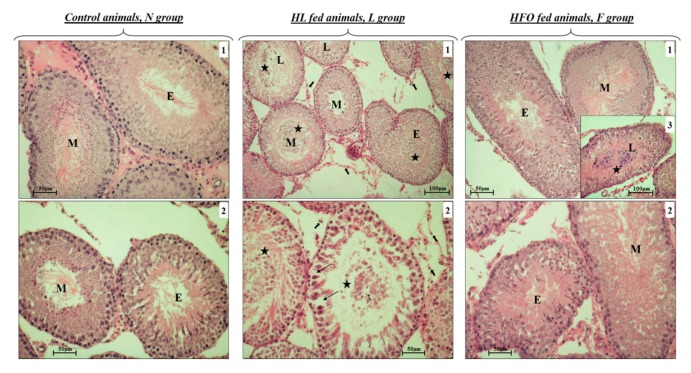
Morphological analysis. Morphological alterations of seminiferous tubules were evaluated using H&E stain of tissular sections. Control animals showed a normal cellular organization in the seminiferous epithelium, with a correct cellular distribution, maturation, and differentiation during sperm production (N group, panels 1 and 2). The effects of both HL and HFO diets showed major alterations in HL-treated animals (L group): Large space in the seminiferous epithelium (panel 2, thin black arrows) where germinal cells were found detached from Sertoli cells; nondifferentiated cells in the lumen in L group (L group, panels 1 and 2, black stars); a certain degree of Leydig cells atrophy was noted (L group, panel 1 and 2, big black arrows). HFO-treated animals (F group) did not present severe morphological alterations compared to N: Nondifferentiated cells were only detected in tubular lumen in few tubules, (F group, panel 3, black star). Magnification used: 10× and 20×. Scale bars applied: 50 µm and 100 µm. E (early stage); M (middle stage); L (late stage).

In N rats, H&E stain evidenced a normal structure and organization of the seminiferous tubules (Figure 2, N, panels 1 and 2). We did not observe cellular disorganization or alterations in spermatogenesis, in accordance with previous data [41].

L rats exhibited several altered tubules. Some of these tubules showed contracted lumina, whereas, in other tubules, lumina were found much more dilatated. In the E, M, and L stages, it was possible to observe germ cells sloughed into the lumen (Figure 2, L, panels 1 and 2, black stars). Tubular wall presented many vacuoles and spaces (Figure 2, L, panel 2, thin black arrows), where it was sometimes possible to observe germinal cells detached from Sertoli cells. Many cells in the wall appeared eosinophilic with pyknotic nuclei, suggesting apoptotic events. Some tubules showed the wall formed by one or two cellular layers due to the exfoliation of the seminiferous epithelium. In addition, in the M stage that corresponds to spermiation, the number of spermatozoa seemed lower in L group than in the other groups. Moreover, we noted spermatids retention in L stages. These cells were retained in the basal cytosol of Sertoli cells, which underwent death. Leydig cells presented a certain degree of atrophy compared to N and F (Figure 2, L, panels 1 and 2, big black arrows).

F rats did not show any significant differences compared to N animals. In some cases, few non-mature cells were found in the tubular lumen, probably due to a mild cell exfoliation (Figure 2, F, panels 3, black star) not always detectable in this animal group.

### 2.2. Different Impact of HL and HFO Diet on Lipid Peroxidation and Antioxidant Enzymes Activities

**Figure 3:**
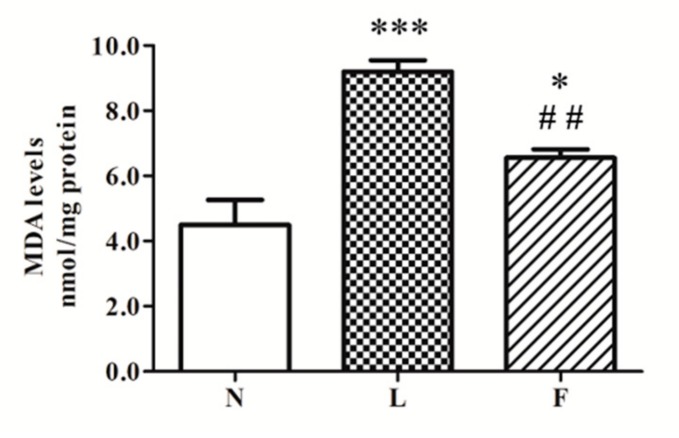
MDA levels. The amount of MDA in total testis homogenate was graphically represented as mean ± SEM of eight different animal for each group. Significant differences are shown: * *p* < 0.05 vs N; *** *p* < 0.001 vs. N; ## *p* < 0.01 vs. L.

A different pro-oxidant effect between HL and HFO diets was evidenced in terms of MDA levels, one of the main products of lipid peroxidation. Our results showed the highest MDA content in L vs. N (~100%) and F (~30%) rats. Moreover, F animals exhibited a level of MDA with a value intermediate between N and L values, with a significant increase (~45%) vs. N (Figure 3).

**Figure 4:**
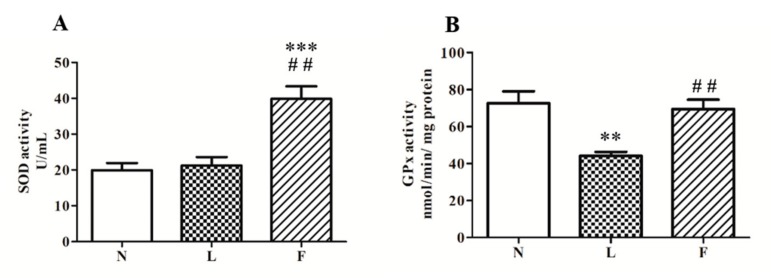
Antioxidant activities. Superoxide dismutase (SOD) (**A**) and glutathione peroxidase (GPx) (**B**) activities were detected on total testis homogenates. Data were graphically represented as mean ± SEM of eight different animal for each group. Significant differences are shown: ** *p* < 0.01 vs. N; *** *p* < 0.001 vs. N, ## *p* < 0.01 vs. L.

The activities of SODs and GPx, the main enzymes involved in redox cellular homeostasis, were found differently modulated by HL and HFO diet. Regarding SODs (Figure 4A), we found a significant increase of enzymatic activity only in F rats vs. N (~100%) and L (~90%) rats. On the contrary, GPx activity level (Figure 4B) was found reduced in L rats (~40%) and unchanged in F vs. N group.

### 2.3. A Possible Link Between Oxidative Stress and Pro-Apoptotic Signals

**Figure 5:**
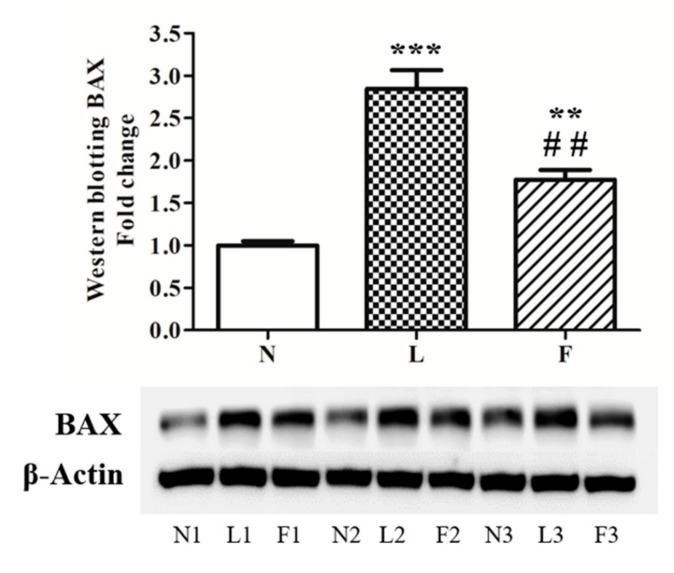
BCL2-associated X (BAX) protein levels. The analyses of BAX protein levels were monitored by western blotting. The figure shows a representative western blot and the densitometric analyses were graphically represented as mean ± standard error on the mean (SEM) of six different animals for each group expressed as fold change vs. N. Significant differences are shown: ** *p* < 0.01 vs. N; *** *p* < 0.001 vs. N; ## *p* < 0.01 vs L.

In accordance with MDA levels, we showed increased BCL2-associated X (BAX) protein levels in high-fat fed animals. The highest BAX protein level was found in L rats compared to N (+180%) and F (+60%), respectively. F rats exhibited a significant increased BAX protein levels vs. N (+80%), whereas this increase was lower than the one found in L rats (Figure 5).

**Figure 6:**
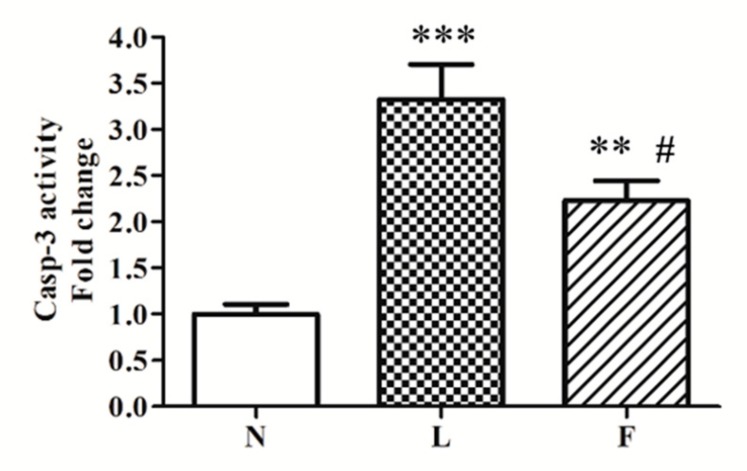
Caspase 3 activity. The analyses of caspase 3 (casp-3) activity was measured by colorimetric assay. Data were graphically represented as mean ± standard error on the mean (SEM) of eight different animals for each group. Significant differences are shown: ** *p* < 0.01 vs. N; *** *p* < 0.001 vs. N; # *p* < 0.05 vs. L.

In line with BAX levels, caspase 3 activity was found increased in L rats vs. N (~3-fold) and F (~1-fold), respectively (Figure 6). On the contrary, F animals exhibited a ~2-fold increase in caspase 3 activity vs. N (Figure 6), but the activity was lower than in L group.

### 2.4. HFO Diet Induced a Shift of Mitochondrial Dynamics Toward Fusion Processes

**Figure 7:**
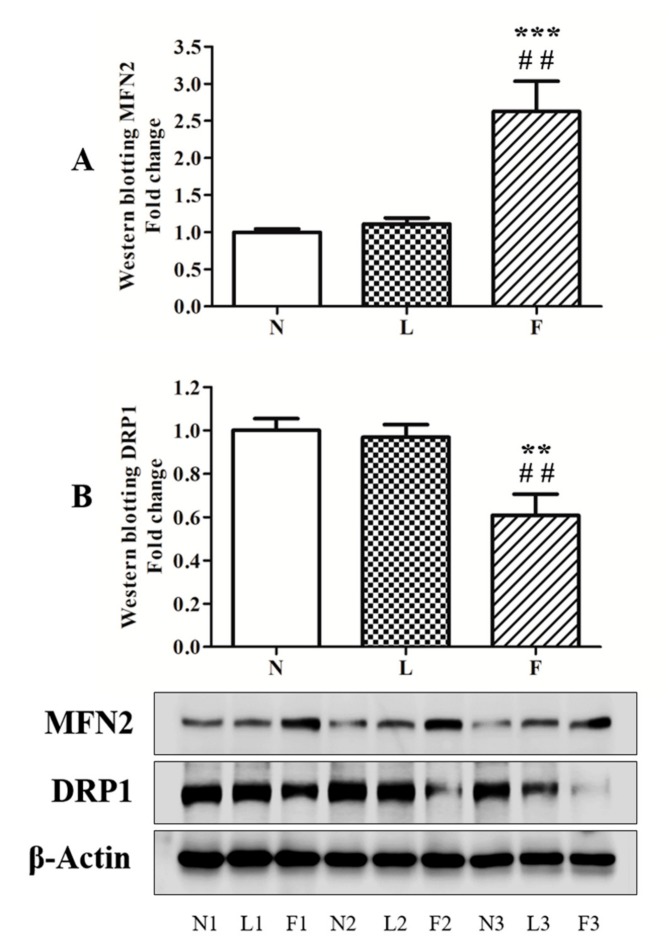
MFN2 (**A**) and DRP1 (**B**) levels. Dynamic proteins contents were monitored by western blotting. The figure shows a representative western blot and the densitometric analyses were graphically represented as mean ± SEM of six different animals for each group expressed as fold change vs. N. Significant differences are shown: ** *p* < 0.01 vs. N; ## *p* < 0.01 vs L.

To evaluate the possible correlation between oxidative injury and mitochondrial dynamic behavior, which directly correlates with mitochondrial morphology and function [42], MFN2 and DRP1, the principal proteins involved in the mitochondrial dynamics, were analyzed by western blotting. Results showed that fusion and fission processes change in different way in L and F rats compared to the control animals. Regarding MFN2 (Figure 7A), no changes in protein contents were observed in L vs. N rats, whereas F animals showed significantly increased (~260%) protein levels compared to both N and L group. On the other hand, DRP1 content did not change between L and N group, whereas significant reduction (~40%) was found in F compared to N and L rats (Figure 7B).

## 3. Discussion

Our study was conducted to evaluate the possible role of mitochondria in the testicular cell adaptation to chronic high-fat feeding with different dietary fat sources. In particular, we tested the effects of two different chronic high-fat dietary regimens characterized by SFA (lard as dietary source of fat) or ω3-PUFAs (fish oil as dietary source of fat) to evaluate testicular histology impairment, oxidative stress, mitochondrial dynamic fusion/fission protein, and stimulation of apoptosis. As mentioned above, our previous work showed that a chronic HFD administration, rich in SFA, induced body weight gain and testicular impairment, such as oxidative stress, morphological alterations, and apoptosis in cells of the seminiferous epithelium, suggesting a negative effect of SFA on testis function [42].

In the present work, both HL and HFO diets induced obesity development, but HL diet showed higher obesogenic effect than HFO diet as shown by body weight gain, confirming our previous data and literature knowledge [24,30,31,43,44]. We also previously reported that HFO diet resulted in a lower increase in serum lipids and cytokines levels than HL diet in the same animal experimental model [30,31].

In addition, our present finding evidenced different responses to the different diet treatments in rat testis. With regard to tissular alterations, we showed severe morphological changes in L rats compared to the other analyzed groups. The images well-evidenced alterations of tubular architecture, with compromising cellular disposition in the seminiferous epithelium and presence of eosinophilic cells. Moreover, many tubules presented the lumina fulfilled with nondifferentiated cells, large spaces between germinal cells, and, in some cases, reduction in thickness of the germinative epithelium with consequent increase in the volume of tubular lumen. All data suggest a possible deleterious effect of HL diet by interrupting the blood-testis barrier with reduction in the tight junction related proteins, as also reported by other authors [45]. On the contrary, HFO diet did not negatively affect testicular morphology at the same extension of HL diet. In fact, F animals presented much more normal tubules with the typical physiological structure, as observed in the N group. However, some tubules with morphological changes were histologically detectable, showing few nondifferentiated cells in the lumina. Cellular disposition in the seminiferous epithelium was not severe compromised. According to histological data, testicular weight in L rats was reduced compared to control animals, whereas any change in testis weight was detected in F.

At molecular level, a different degree of oxidative injury was detected in L and F groups, with different response in antioxidant defense, mitochondrial dynamic behavior, and apoptotic onset. Cellular oxidative damage is the result of imbalance between ROS levels, principally produced by mitochondria, and antioxidant cellular response. Therefore, in a contest of mitochondrial ROS overproduction, cells try to reorganize their metabolism to quench radical species, for example, by stimulating antioxidant system activities. Moreover, mitochondria respond to different degree of cellular stress remodeling their structure by changing fusion/fission cycles to eliminate or recover part of mitochondria or to direct the cells toward death [46].

Our results showed the highest MDA levels in total homogenates in L rats compared to the other groups, suggesting a greater lipid oxidative damage. In fact, the excess of MDA reelevates lipid peroxidation in cell membranes. Moreover, reduced GPx activity was detected in L rats, suggesting a possible imbalance between ROS accumulation and antioxidant capacity. It is worth noting that F animals showed MDA levels intermediate between N and L rats associated with increase in total SOD activity vs. N and L group. These data suggested that dietary SFA play a major pro-oxidant role than ω3-PUFAs. The lower MDA levels in F vs. L could be due to the increased SOD activity, useful to control superoxide anion produced by mitochondria. One of the members of SOD family is the antioxidant enzyme SOD2 (Mn-SOD), which plays its antioxidant role at mitochondrial level. SOD2, in fact, localizes in the mitochondrial matrix, and it detoxifies the superoxide anion, abundantly produced during mitochondrial respiration to H_2_O_2_. This molecule is converted to H_2_O by GPx activity [47]. In this way, antioxidant system counteracts ROS production and oxidative damage. However, when the ROS production rate in the cells is not completely balanced by antioxidant defenses, oxidative damage occurs on several macromolecules altering cellular functions. Therefore, antioxidant activity represents a key role in cellular redox homeostasis and mitochondria are directly involved.

As described above, in our investigation, we evidenced tubular alterations especially in L group, where the highest antioxidant impairment and oxidative injury was observed. For these reasons, we decided to analyze the pro-apoptotic effects of both HF and HFO diets by testing BAX protein content in total homogenates. This pro-apoptotic marker functions as mitochondrial target [48,49]. In fact, at the mitochondrial level, BAX generates outer membrane permeabilization, and cytochrome c release from mitochondrial inner membrane space to cytosol, inducing apoptosis activation.

In line with increased BAX protein levels, caspase 3 activity increased in total homogenates in high-fat fed animals with the highest levels in L group. These data suggested that ω3-PUFAs could play a positive role in the control of apoptosis. In this contest, mitochondrial performance appears to be a crucial point in the regulation of this physiological processes. All data produced in this work indicated that SFA and ω3-PUFAs differently affect testicular antioxidant capacity, apoptosis, and tissue morphology, suggesting a possible mitochondrial implication to contribute to these differences. Given that it is known from literature that SFA and ω3-PUFA are able to change mitochondrial fusion/fission processes, we evaluated if there was a change in mitochondrial dynamic behavior in testis. The analyses of DRP1 (fission marker) and MFN2 (fusion marker) content showed that the HL diet did not produce any mitochondrial dynamic changes compared to the control diet. It is worth noting that F animals showed significant reduction in DRP1 levels associated with increased MFN2 levels. These data suggest that HFO diet induced a shift of mitochondrial dynamics toward fusion process in testis, in accordance with data reported in other tissues [24,30]. With the limitation that other mitochondrial dynamic proteins should be evaluated and considering that HFO diet mainly differentiated by HL diet for the higher ω3-PUFA vs. SFA content, the induction of mitochondrial fusion process may represent a possible link between ω3-PUFA and mechanisms used by cells to improve mitochondrial function and network in order to control cellular stress and death.

## 4. Materials and Methods

### 4.1. Ethics Statement

This study was conducted in accordance with recommendations in the EU Directive p2010/63/ for the Care and Use of Laboratory Animals. The protocol was approved by the Committee on the Ethics of Animal Experiments of the University of Naples ‘‘Federico II’’ (Permit Number: 2010/0149862, 16 December 2010).

### 4.2. Materials

The chemicals to compose all buffers used for the experiments were purchased from Sigma Aldrich (St. Louis, MO, USA). The fish oil used to feed “F” animals was cod liver oil (New.Fa.Dem. srl, Giugliano, Naples, Italy).

### 4.3. Experimental Design

Male Wistar rats, age 60 days (Charles River Italia, Calco, Como, Italy), were caged separately in a temperature-controlled room at 24 °C with a 12 h light-dark cycle. After one week of acclimation, rats were divided into three experimental groups (eight rats for each group) with a similar mean body weight (approximately 400 g) and with the body weights normally distributed within each group. The first group (N rats) received a standard diet (10.6% fat J/J); the second group (L rats) received a high-fat diet rich in lard (HL diet,40% fat J/J); the third group (F rats) received a high-fat diet rich in fish-oil (HFO diet,40% fat J/J). The animals were fed ad libitum and the period of treatment lasted six weeks. Diet compositions are shown in Table 1. The two high-fat diets were formulated to differ from the standard low-fat diet with regard to the contributions of fat and carbohydrate to the energy value but to be identical in terms of proteins, vitamins, minerals, and fibers. The two high-fat diets have the same fat energy content, but they differ in the source of fat. In fact, HL diet was obtained using lard as source of saturated fatty acids (SFA), whereas HFO diet was obtained by using fish oil as source of ω-3 polyunsaturated fatty acids (ω3-PUFAs). The body weights were monitored daily to enable calculations of body weight gain.

At the end of the experimental period of six weeks, the rats were anesthetized by an intraperitoneal injection of Zoletil (40 mg/Kg body weight) and euthanized by decapitation. Testicles were immediately removed, weighed, and processed in accordance with the experimental procedures used. Testis slices were either immediately processed for morphological analysis or frozen in liquid nitrogen and stored at −80 °C for later processing.

### 4.4. Morphological Analysis

After euthanasia, one testicle for each animal was immediately removed, washed in 0.9% NaCl, and fixed in Bouin’s fluid solution. Then, each testicle was dehydrated in ethanol, included in paraplast and sliced at 5 µm using a microtome. Morphological analysis was performed using haematoxylin and eosin stain and the images were acquired with a Zeiss Axioskope light microscope fitted with a TV camera. To describe testis morphology, the tubules were differentiated based on spermatogenic cycle of the seminiferous epithelium in early (E, I-VII), middle (M, VIII), and late (L, IX-XIV) stages.

### 4.5. Lipid Peroxidation

Lipid peroxidation was evaluated measuring the amount of MDA accumulated in the tissue using thiobarbituric acid reactive substances (TBARS) assay kit (Cayman Chemical Company, No: 10009055). About 25 mg of tissue were washed in cold ice PBS (1.4 mM KH_2_PO_4_, 8 mM Na_2_HPO_4_, 140 mM NaCl, 2.7 mM KCl, pH 7.4), homogenate in 250 µL of RIPA Buffer solution (150 mM NaCl, 50 mM Tris pH 7.4, 1% Triton X-100, 0.5% sodium deoxycholate, 0.1% SDS) containing a cocktail of protease inhibitors, centrifuged at 1600× *g* for 15 min at 4 °C, and the obtained supernatant were processed in accordance with the kit. Protein content was evaluated by the Hartree method [50] using BSA to produce a standard curve. MDA concentration was calculated as indicated by manufacturer, represented graphically as fold change vs. N and expressed as nmol MDA per mg of proteins.

### 4.6. Antioxidant SOD and GPx Activities

To determine both SOD and GPx activities in the total homogenates, standard commercial colorimetric kits from Cayman Chemical Company were used. Total SOD activity was monitored by using kit No.706002. Total GPx activity was evaluated by using kit No. 703002.

### 4.7. Electrophoresis and Western Blotting

After euthanasia, slices of testis were used to obtain total protein extract for western blotting analysis. Briefly, 150 mg of tissue were homogenated in 1 mL of RIPA Buffer solution containing protease inhibitors. The homogenate was obtained using a polytron (KINEMATICA Polytron Model PT10-35 GT/PT 3100D Homogenizer, Fisher Scientific) and centrifuged at 12,000× *g* for 15 min. Supernatant was analyzed to calculate protein concentration in each sample and used for western blotting procedure. About 30 µg of proteins were electrophoresed on SDS-polyacrylamide gels as described by Laemmli [51] together with a standard protein marker (Color Burst Electrophoresis Marker, m.w. 8000–220,000 Da). After running, proteins were transferred onto nitrocellulose membrane Immobilon-P, Millipore) at 350 mA for 1 h. Then, membranes were blocked in blocking solution (TBS + 0.1% Tween-20 + 5% milk) for 1 h at room temperature and incubated O.N. with the primary antibodies of interest: DRP1 (rabbit polyclonal antibody, sc-32898, 1:500); MFN 2 (mouse monoclonal antibody, sc-100560, 1:500); BAX (rabbit polyclonal antibody, sc-526, 1:200); ß-Actin (mouse monoclonal antibody, sc-70319, 1:200).

On the second day, membranes were washed in TBS-Tween solution (TBS + 0.1% Tween-20) and incubated with the appropriated secondary antibodies labelled with horseradish peroxidase (donkey-anti rabbit, IgG-HRP: sc-2313, 1:5000 or goat-anti mouse, IgG-HRP: sc-2005, 1:5000) in TBS-Tween + 5% milk for 1 h at room temperature. Again, membranes were washed in TBS-Tween solution and the immunoreactive bands were obtained by using luminol reagent. Bands signal intensity was analysed with a C-DiGit Chemiluminescent Western Blot Scanner (LI-COR). ß-actin was used as loading control guide.

### 4.8. Caspase 3 Activity

Caspase 3 activity was analyzed in total homogenate using a colorimetric kit from Sigma Aldrich (CASP3C-1KT).

### 4.9. Statistical Analysis

Statistical analyses were carried out with Graph Pad software and shown as fold change of the mean ± standard error on the mean (SEM) vs. control animals. Differences between groups were analyzed using one-way ANOVA followed by Bonferroni Post-hoc test. Statistical differences were considered significant when *p* value was inferior to 0.05.

## 5. Conclusions

In conclusion, our studies showed that SFA and ω3-PUFAs chronic overfeeding determined different effects on testis by generating cellular responses dependent on dietary fat quality. The most harmful effects on testicular health were found in L rats, suggesting that the excess of dietary SFA can negatively act on reproduction. On the contrary, mitochondrial dynamics variation toward fusion process detected in F group could suggest a possible mechanism by which ω3-PUFAs elicited lower dangerous effects compared to SFA. The novelty in the present report was that mitochondrial fusion/fission proteins may be considered as target of bioactive compounds, such as ω3-PUFA, to induce improvement in mitochondrial function and reduce oxidative stress in association with amelioration in antioxidant defense in order to maintain testis function during chronic overfeeding. Moreover, the present work opens a new perspective in clinical nutrition, and it can be speculated that an adequate ω3-PUFAs intake may represent a nutritional goal in our society to counteract the negative effects of high SFA intake not only on cardiovascular and metabolic disease, but also on testis function, by targeting mitochondria. However, further analyses are needed to highlight the effects of fatty acids on mitochondrial functionality and to describe the sequence of events in which ω-3 PUFA are involved to control cell damage while focusing on the role of mitochondria in the regulation of testicular health.

## Figures and Tables

**Table 1:** Diet composition.

Component	Control Diet	High-Fat Diet	
		High lard (HL) g/100 g	High fish oil (HFO) g/100 g
Standard feed (g)	100	51.03	51.03
Casein ^1^ (g)	-	9.25	9.25
Lard (g)	-	21.8	-
Fish oil ^2^ (g)	-	-	21.8
Sunflower oil (g)	-	1.24	1.24
AIN 76Mineral mix ^3^ (g)	-	1.46	1.46
AIN 76Vitamin mix ^4^ (g)	-	0.42	0.42
Choline bitartrate (g)	-	0.08	0.08
Methionine (g)	-	0.12	0.12
Energy density (KJ/g diet)	15.88	20.0	20.0
Protein %	20.0	29.0	29.0
Lipid %	10.6	40.0	40.0
Carbohydrates %	60.4	31.0	31.0

^1^ Purified high-nitrogen casein containing 88% protein; ^2^ Fish oil = The fish oil used was cod liver oil (New.Fa.Dem. srl, Giugliano, Naples, Italy) containing vitamin A (50–500 UI/g; 15–150 μg) and vitamin D3 (50 U.I./g; 1.3 μg); EPA ≈ 722 mg/Kg body weight/die and DHA ≈ 1153 mg/kg body weight/die; ^3^ American Institute of Nutrition (1977); ^4^ American Institute of Nutrition (1980).
